# Oral Lipoma Resembling Popeye's Pipe: A Case Report

**DOI:** 10.7759/cureus.22350

**Published:** 2022-02-17

**Authors:** Vural Akın, Erdoğan Okur, Yusuf Ç Kumbul, Nazan Okur, Rabia Kum

**Affiliations:** 1 Department of Otorhinolaryngology and Head and Neck Surgery, Faculty of Medicine, Suleyman Demirel University, Isparta, TUR; 2 Department of Radiology, Faculty of Medicine, Suleyman Demirel University, Isparta, TUR; 3 Department of Pathology, Faculty of Medicine, Suleyman Demirel University, Isparta, TUR

**Keywords:** excision, buccal area, geriatrics, oral cavity, lipoma

## Abstract

Lipomas are benign neoplasms of mesenchymal origin. Although they are frequently seen in other parts of the body, they are rare in the oral cavity. In the oral cavity, they most often develop from buccal mucosa. They tend to grow slowly, so they may remain asymptomatic for a long time and go unnoticed. Lipomas in the oral cavity may cause deterioration in chewing-speaking and esthetic problems over time, depending on the increase in their size. The most reliable imaging method for differential diagnosis is magnetic resonance imaging. Complete excision of the lipoma is essential for treatment. In this study, a case of an unusual oral lipoma, causing nutrition-speaking difficulties in a geriatric male patient is presented.

## Introduction

Lipomas are benign mesenchymal neoplasms that can be seen throughout the body, popularly known as the "universal tumor" because of its ubiquitous presence anywhere in the body [1-4]. They are rarely observed among all oral cavity benign lesions with the incidence of 1-4.4% [2,4,5]. While there is no difference in frequency between genders, the incidence increases over the age of 40 years [4,6-8]. Lipomas most commonly develop from the buccal mucosa of the oral cavity and tend to grow slowly [4,5]. These lesions, which grow painlessly in the mouth, are usually surgically removed when they cause esthetic problems [5,6]. The recurrence rate is less than 5% if the capsule is excised without disrupting its integrity [5]. In this study, a case of lipoma that protruded out of the mouth and reached a relatively large size, causing nutrition and speech impairments, is presented in a geriatric patient.

## Case presentation

An 87-year-old male patient was admitted to the otorhinolaryngology clinic because of a mass originating from the inside of the mouth and protruding from the mouth. The patient had noticed the mass on his left cheek about 20 years ago, and this mass had grown over time. He stated that the mass was not painful, but recently he had difficulty in chewing and speaking because of the mass. For this reason, he said that his nutrition deteriorated and weight loss occurred. The patient had no additional disease other than benign prostatic hyperplasia and had no history of chronic drug use. The patient had no history of intraoral surgery.

**Figure 1:**
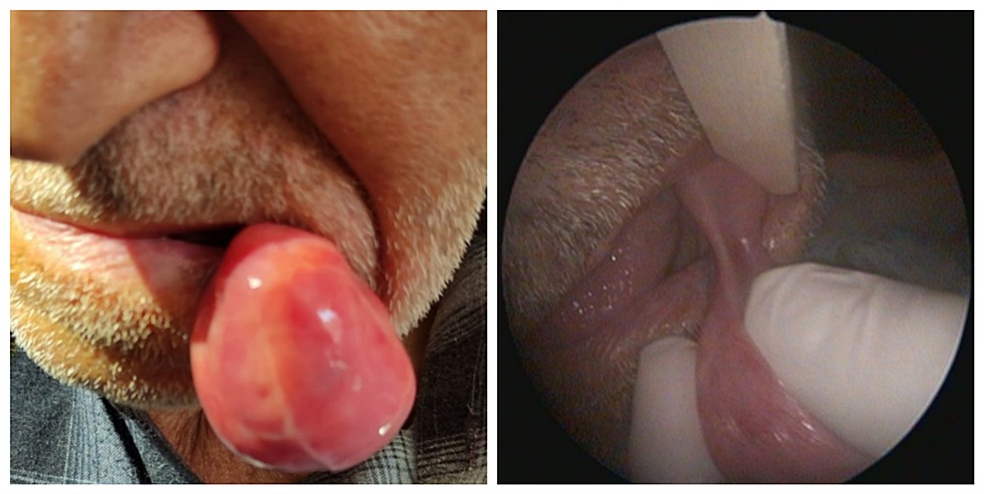
Image of the mass at the time of first admission.

**Figure 2:**
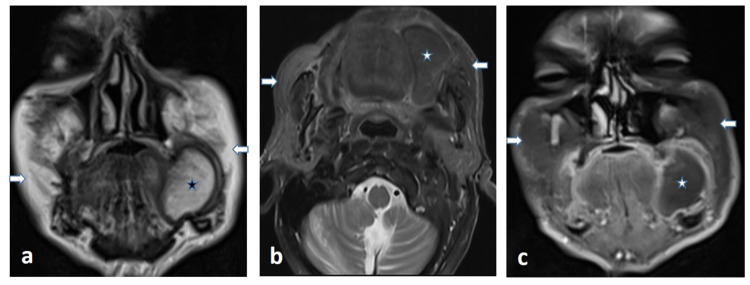
MRI images of the maxillofacial region. In the coronal T2W image (a) the well-circumscribed mass lesion (asterisk) in the right buccal region is completely isointense to the subcutaneous fatty tissue (arrows). Axial fat-suppressed T2W image (b) demonstrates total signal loss in both the lesion (asterisk) and also subcutaneous fatty tissues (arrows) which is consistent with the mass’ lipomatous nature. Contrast-enhanced coronal fat-suppressed T1W image (c) shows that there isn’t any enhancement of the lipomatous mass except for its peripheral thin smooth capsule. Lipoma is diagnosed as per the findings. T2W: T2-weighted; T1W: T1-weighted

On physical examination, it was observed that he used removable total dentures for the lower and upper jaws. On the left buccal mucosa, a mass of approximately 5x5 cm, with a pedicle of approximately 1 cm in diameter, with regular mucosa, soft consistency, pink-yellow color was observed just lateral to the maxillary first and second premolar teeth (Figure 1). The patient's complete blood count and biochemical parameters were within normal limits. The mass was evaluated by contrast-enhanced magnetic resonance imaging (MRI) which clearly demonstrated the lipomatous nature of the lesion (Figure 2).

**Figure 3:**
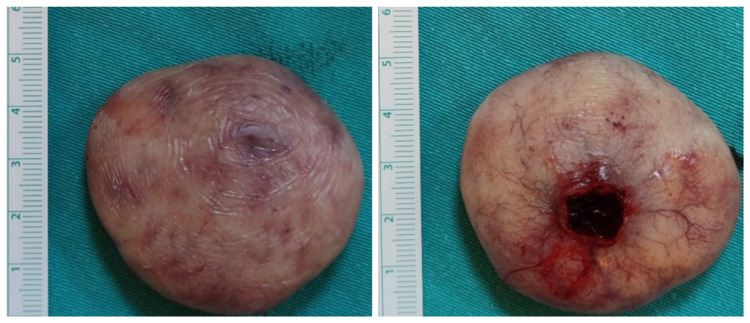
Image of the mass after excision.

**Figure 4:**
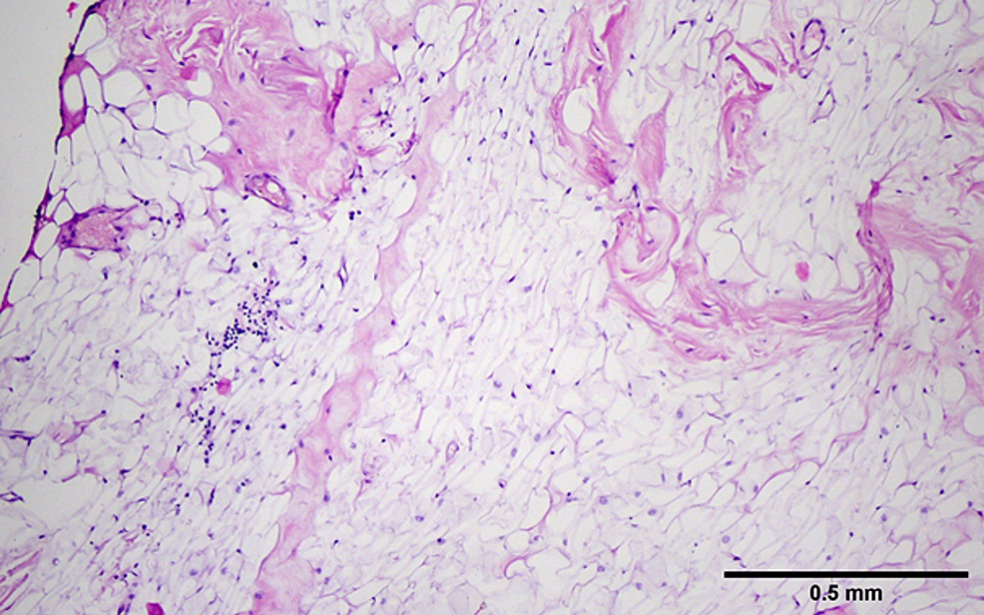
Histopathological examination image of the lesion. The image shows lipoma developing from mature adipose tissue, containing thin collagen bundles, with a thin capsule structure visible on the left (100x, hematoxylin and eosin).

It was decided to excise the mass under local anesthesia (Figure 3). The patient was discharged on the same day with recommendations about oral hygiene. Histopathological examination of the mass reported lipoma (Figure 4). No recurrence was observed in the sixth month of follow-up.

## Discussion

The reason why the term ‘universal tumor’ is used for lipomas is that they can occur anywhere in the body. The head and neck region is one of these areas, and lipomas in this region constitute 15-20% of all lipomas [1,9]. Oral lipomas are rare benign lesions in the oral cavity [2,4]. Although in the oral cavity they are most commonly located in the buccal area, other locations include the lips, palate, salivary glands, tongue, and floor of the mouth [5]. The case we presented developed from the buccal mucosa, which is the most common area of involvement of oral lipomas in literature. We think that the reason for the frequent involvement of the buccal area is the fatty pads in this area.

Trauma, infection, hormones, and genetic causes are suspected in the formation of lipomas whose etiology is not clearly known [10]. In addition to the aforementioned suspicious causes, it has been also shown that oral lipoma might be associated with human papillomavirus-16 (HPV-16) [11]. Lesions such as fibroma, schwannoma, neurofibroma, dermoid and epidermoid cysts, salivary gland tumors, foreign body granuloma, traumatic neuroma, and hemangioma should be considered in the differential diagnosis of oral lipomas [3,8,12]. Initially, we diagnosed traumatic fibroma first in the differential diagnosis of this case because we thought that the patient's use of an intraoral prosthesis could trigger the formation of traumatic fibroma. In addition, oral lipomas are usually yellow in color [5]. The lesion in this patient looked pink-yellow in color. The color of the lesion also led us to consider traumatic fibroma. In short, the most important risk factor for lipoma formation in our case seemed to be trauma-related to removable total dentures.

Oral lipomas can be seen at any age and they are more common in individuals over the age of 40 and tend to grow slowly [4,6]. Since they increase in size over time, they may cause difficulties in chewing and speaking functions and esthetic problems [5,6]. Our patient had a history of oral lipoma for about 20 years. He had lost 7 kilograms in the last six months. Especially in geriatric patients, electrolyte imbalance and dehydration, which can be fatal, may occur due to such lesions in the mouth. Therefore, we recommend that oral cavity lesions that cause nutritional problems in geriatric patients should be excised as soon as possible.

For treatment, it is sufficient to excise the lipoma without disrupting the capsule integrity, and it usually does not require aggressive excision and additional treatments [5]. We excised the lipoma in our patient under the conditions mentioned and the case did not have recurrence during the sixth-month follow-up.

## Conclusions

Lipomas should be included in the differential diagnosis of lesions originating from the buccal mucosa of oral cavity. Lipomas with benign character and slow growth patterns can reach extraordinary sizes. We recommend that they should be excised as soon as possible, especially in geriatric patients, as they can cause nutritional problems.
